# War Neuroses

**Published:** 1918-08

**Authors:** 


### NEUROLOGY AND PSYCHIATRY

***War Neuroses.*** By Wm. B. Terhune, 1st Lieut. M. R. C., U. S. A. *Journal of the American Medical Association*, May 11, 1918.

*Nomenclature* : As there is no definite knowledge of the neurologic pathology and as the psychologic processes are largely surmise, numerous terms have been suggested. Eder makes use of the term " War Strain ". Babinski has originated the word " pithiatism " to describe a hysteroid state in which the symptoms are the result of suggestion and may be relieved by suggestion. All other forms of war neuroses he has designated as " reflex nervous disorders " and he believes they are on the border line between functional and organic disease.

British Medical officers consider the disease as purely functional and employ the terms " shell shock " and neurasthenia — the differentiation depending on whether or not the individual has been exposed to extraordinary strain. Shell shock as a diagnosis has resulted in an increase in the prevalence of the disease — chiefly because of this the name is not used until the patient has been carefully examined in a hospital especially for nervous disorders and not then until information has been obtained from his unit verifying his own statement as to how his illness arose. Malingerers will attempt to imitate this condition when it is remembered that many men at the front suffer from mild forms of it and that a man who wishes to shirk his duty may easily exaggerate these mild symptoms.

*Predisposing Causes* : To suffer from shell shock does not necessarily stigmatize an individual as neurotic, although many of these patients have suffered in civil life from various forms of mental instability. Landenheimer states that 90 o/o were predisposed before joining the army — Forsythe 100 o/o — Mott 66 o/o — Eder 30 o/o. However, some of the most courageoüs men apparently free from all neurotic tendencies are affected, The disturbance occurs among commissioned officers, non commissioned officers and privates — a relatively larger percentage amongst the non-commissioned officers and yet they are the men chosen especially for their bravery and dependability.

The men are constantly under a strain, physical and mental

For several miles behind the line there is no place of safety, and sleep is often out of the question. The knowledge of bombing experiences that may be repeated at any time does not tend to increase mental composure — the men often continue to dodge shells after being admitted to a hospital, so vivid is the memory to them. Fatigue is also a very important factor. It is very doubtful if psychoneuroses occur as the result of fatigue unless associated with intense emotion. Fear is one of the strongest of the emotions and while it may be repressed, nevertheless it exists and exerts its influence.

*Symptoms* : Headaches are probably the most constant symptom, especially in the occipital region. Insomnia is quite common. Cyanosis of the hands is noticeable. About 1/4 of the patients have tachycardia.

*Treatment* : Military discipline is never relaxed. Drugs are of very little use. Verinal is given for insomnia — phenacetin for headache. In acute cases, several hours in a hot bath seem to be more beneficial than anything else. Mild cases are practically well in a few days and return to their units. A man is in better condition to return to his unit after a month on a farm than after the same length of time in a convalescent dépôt.

